# Reduning Attenuates LPS-Induced Human Unmilical Vein Endothelial Cells (HUVECs) Apoptosis Through PI3K-AKT Signaling Pathway

**DOI:** 10.3389/fphar.2022.921337

**Published:** 2022-07-12

**Authors:** Ziyi Wang, Xuesong Wang, Zhe Guo, Haiyan Liao, Yan Chai, Ziwen Wang, Zhong Wang

**Affiliations:** ^1^ School of Clinical Medicine, Tsinghua University, Beijing, China; ^2^ Department of Liver Intensive Care Unit, Beijing Tsinghua Changgung Hospital, Beijing, China

**Keywords:** sepsis, reduning, PI3K, akt, apoptosis

## Abstract

The molecular mechanism of Reduning (RDN) in the treatment of sepsis was analyzed based on network pharmacology. The system pharmacology method was administered to search the active ingredients and targets of RDN, identify the sepsis-related genes, and determine the targets of RDN in the treatment of sepsis. Cytoscape was used to build a “drug component-target” network to screen key compounds. A protein-protein interaction (PPI) network was constructed using STRING, and core targets were revealed through topological analysis. 404 shared targets of RDN and sepsis were introduced into DAVID Bioinformatics Resources 6.8 for GO and KEGG enrichment analysis to predict their possible signaling pathways and explore their molecular mechanisms. GO enrichment analysis highlighted that they were largely related to protein phosphorylation, inflammatory reaction, and positive regulation of mitogen-activated protein kinase (MAPK) cascade. KEGG enrichment analysis outlined that they were enriched in PI3K-AKT signaling pathway, calcium signaling pathway, rhoptry-associated protein 1 (Rap1) signaling pathway, and advanced glycation end products and receptors for advanced glycation end products (AGE-RAGE) signaling pathway. Molecular biological validation results exposed that RDN could significantly improve the protein expression of p-AKT and p-PI3K, alleviate apoptosis-related proteins expression level and decrease apoptosis rate in LPS-induced HUVECs. In conclusion, it was illustrated that RDN could considerably constrain LPS-induced apoptosis by activating the PI3K-AKT signaling pathway, which advocated a basis for fundamental mechanism research and clinical application of RDN in the treatment of sepsis.

## 1 Introduction

Sepsis is a common disease in critically ill patients. Despite significant advances in early diagnosis and organ function support in patients with sepsis over the past few decades, sepsis continues to have high morbidity and mortality rates (Xie et al., 2020; Bruno et al., 2021). Anti-infection, dilation, diuresis, the use of vasoactive drugs, and hemodialysis are the main treatments which could save lives and reduce mortality with sepsis (Jarczak et al., 2021). But their efficacy is still not ideal due to the lack of early diagnosis and the large differences among patients (Scheer et al., 2019). Traditional Chinese medicine (TCM) has dramatically shaped queries on sepsis in recent years (Wang et al., 2021; Zhou et al., 2021). RDN injection is a kind of TCM injections, which was composed of Artemisia annua, Honeysuckle and Gardenia gardenia (Jiang et al., 2019). With the effects of clearing heat, dispersing wind and detoxifying, RDN is widely accepted as the treatment of acute infectious disease (Zhang et al., 2013; Luo et al., 2022). Prior investigations have implemented that RDN could improve the prognosis in early sepsis though reducing the degree of inflammation and improving prognosis (Zhou et al., 2016; Zheng, 2019a). However previous study failed to clarify the molecular mechanism of RDN in the treatment of sepsis. In view of the multi-component, multi-target, and multi-pathway characteristics of TCM, this study explored the molecular mechanism of RDN injection in the treatment of sepsis from the perspective of network pharmacology, in order to provide scientific basis for further research. The research results are reported as follows.

## 2 Data and Methods

### 2.1 RDN Composition and Targets

The corresponding active components of Artemisia annua, Honeysuckle and Gardenia jasminoides were searched using the pharmacologic database and analysis platform of TCM System (TCMSP, https://tcmspw.com/tcmsp.php/). Using oral bioavailability (OB)≥30% and drug-like activity (DL)≥0.18 as screening conditions, a total of 49 active ingredients were screened. We used the Pubchem database (https://pubchem.ncbi.nlm.nih.gov/) to retrieve active TCM ingredients, and then put simplified molecular input line entry system (SMILE) numbers into SwissTarget Prediction platform to acquire targets of TCM.

### 2.2 Targets of Sepsis

Using GeneCards human genome database, DisGenet database (https://www.disgenet.org/) and OMIM database (https://omim.org/), target identification was conducted with “Sepsis” and disease target genes were obtained.

### 2.3 Key Targets of RDN for the Treatment of Sepsis

Venny 2.1 online software mapping tool platform was used to detect key targets of RDN for the treatment of sepsis. The intersection of the two was obtained to be critical targets of RDN for the treatment of sepsis.

### 2.4 Establishment and Analysis of TCM - Component-Target-Sepsis Network

Through screening by TCMSP and BATMAN data library, and with the help of UniProt, we corrected the gene name corresponding to the target with the species limited to “Homo sapiens”. Cytoscape 3.7.2 software was applied to establish a network of “TCM-component-target-sepsis”. The Network analyzer function was employed to analyze the main active ingredients of TCM compounds, and the topology analysis of the network diagram was carried out.

### 2.5 PPI Network Construction and Key Target Screening

The PPI network was constructed by inputting the common targets of drug and disease into STRING database (https://string-db.org/cgi/input.pl), and the species was set as “Homo sapiens” to obtain the PPI network. Then, TSV files obtained from STRING database are imported into Cytoscape software to conduct topological analysis of PPI network and screen out key target genes of compounds acting on diseases according to degree value ranking.

### 2.6 TCM—Compound - Target Network Construction

In order to better understand the complex interaction among TCM, compounds and corresponding targets, a network diagram of TCM compounds and shared targets of RDN and sepsis were constructed, and Cytoscape software was responsible for visualizing.

### 2.7 GO and KEGG Enrichment Analysis

Shared targets of RDN and sepsis were imported into DAVID Bioinformatics Resources 6.8 (https://david.ncifcrf.gov/home.jsp) to analyse GO enrichment, including biological processes (in the process, BP), cell Component (CC) enrichment, and molecular function (MF). *p*-value Cutoff = 0.05, q-value Cutoff = 0.05, the rest defaults to original settings. The shared targets were imported into DAVID Bioinformatics Resources 6.8 to clarify KEGG pathway enrichment. We chose the top 10 pathway according to *p*-value and employed microbioinformatics (http://www.bioinformatics.com.cn/) to visualize.

### 2.8 Molecular Docking Analysis

The compound name, molecular weight and 3D structure of the active ingredients were determined from PubChem database, and the corresponding 3D structure of the active ingredients were downloaded from RCSB PDB database (http://www.rcsb.org/). Then, AutoDock software was administered to prepare the ligands and proteins required for molecular docking. The crystal structure of the target protein was removed water molecules, hydrogenated, modified amino acids, optimized energy and adjusted field parameters, and then the low energy conformation of the ligand structure was satisfied.

### 2.9 Molecular Biological Validation

#### 2.9.1 Reagents

RDN was from Jiangsu Kanion Pharmaceutical Co, Ltd (Jiangsu, China). Goat anti-mouse IgG second antibody, goat anti-rabbit second antibody, anti-cleaved-Caspase-3, and anti-PI3K-AKT signaling pathway antibody were from Abcam (United States). fluorochrome-conjugated secondary antibody was from Biosharp (China). Annexin V -FITC/7-AAD Apoptosis Kit, 10% fetal bovine serum, and Cell Counting Kit were from Beyotime (China).

### 2.9.2 Cell Culture

Human Umbilical Vein Endothelial Cells (HUVECs) cultivated by Laboratory of Tsinghua Changgung Hospital were cultured in an Incubator (SANYO, Japan) under standard Conditions (37°C, 5% CO2). The experiments were performed after two passages. Cells were cultured in Dulbecco’s ModifIed Eagle Medium (DMEM), high glucose (Gibco, United States) containing.

#### 2.9.3 Flow Cytometry Analysis

Cell apoptosis level was probed by flow cytometry. HUVECs were washed twice with phosphate-buffered saline (PBS) and resuspended in 100 µl of 1×binding buffer mixed with 2.5 µl of annexin-V–FITC and 2.5 µl of 7-AAD staining solution for 15 min in the dark at room temperature. And then, with 400 µl additional binding buffer added into mix, the cells were finally evaluated using a flow cytometer (BD, United States). 7- AAD and annexin-V assay Q2 + Q3 were used to perform the apoptosis rate.

#### 2.9.4 Western Blot

Total protein was extracted from cells using the cell lysate and placed on a shaker for 15 min for full contact at 4°C. Samples were electrophoresed in 10% SDS-Page gel and electrophoresis transferred onto a polyvinylidene fluoride membrane. Then, the membrane was blocked in 5% dried milk at 4°Covernight. After centrifugation, The protein concentrations were quantified using the BCA Protein Assay kit (Beyotime, China). The membrane was sealed with milk powder and then incubated with primary antibody at 4°C, followed by incubation with goat anti-mouse IgG secondary antibody for luminescence.

#### 2.9.5 CCK-8 Assay

Cell viability was detected by Cell Counting Kit-8 following protocols. Cells were seeded and cultured into 96-well microplates with a density of 2×10^4^/well in 100 μl medium. Then, the cells were stimulated by LPS (and RDN). After treating for certain (6 h/12 h/24 h) hours, 10 μl of CCK-8 reagent was added to each well and then cultured for 2 h. All experiments were performed in triplicate. We applied wells without cells as blanks. The proliferation of cells was expressed by the absorbance. The absorbance was analyzed at 450 nm using a microplate reader (Bio-Rad, United States).

### 2.9.6 Immunofluorescence

The pretreated cover glasses which cells inoculated into were removed after the cells were nearly monolayer, and then washed twice with PBS. The cells were fixed in 4% paraformaldehyde for 15 min and then permeabilized in 0.3% Triton X 100 for 30 min. The cells were blocked with 5% bovine serum albumin (Sigma-Aldrich) for 30 min at 37°C and exposed to the primary antibody overnight at 4°C. The next day they were washed in PBS and then incubated for 60 min at room temperature with species-appropriate fluorochrome-conjugated secondary antibody. Images were acquired using an Olympus camera and matched software. The operator and analyzer were blinded to the experimental condition and quantified with ImageJ.

#### 2.9.7 Data Processing and Statistical Analysis

SPSS 22.0 (United States) was used to interpret all statistical analysis. Statistical analysis was completed with the data from three independent experiments. *t*-test was used for the comparisons between two groups. ANOVA was used to make comparisons between

Multiple groups. Data are presented as mean±standard deviation (SD), *p*-value < 0.05 was regarded with statistical significance. Furthermore, Prism 8 (GraphPad Software, United States) was responsible for visualizing.

## 3 Results

### 3.1 Active Ingredients and Targets of RDN

The corresponding active components of Artemisia annua, Honeysuckle and Gardenia jasminoides were searched using the pharmacologic database and analysis platform of TCM System. Using OB ≥ 30% and drug-like activity DL ≥ 0.18 as screening conditions, a total of 49 active ingredients were screened. We used the Pubchem database to retrieve active RDN ingredients, and then put SMILE numbers into SwissTarget Prediction platform to acquire 782 targets of RDN (supplementary data).

### 3.2 Targets of Sepsis

Using GeneCardard human genome database, DisGenet database and OMIM database, with “Sepsis” as a search term targets for retrieval, we acquired 3402 sepsis targets (supplementary data).

### 3.3 Key Targets of RDN in the Treatment of Sepsis

**FIGURE 1:**
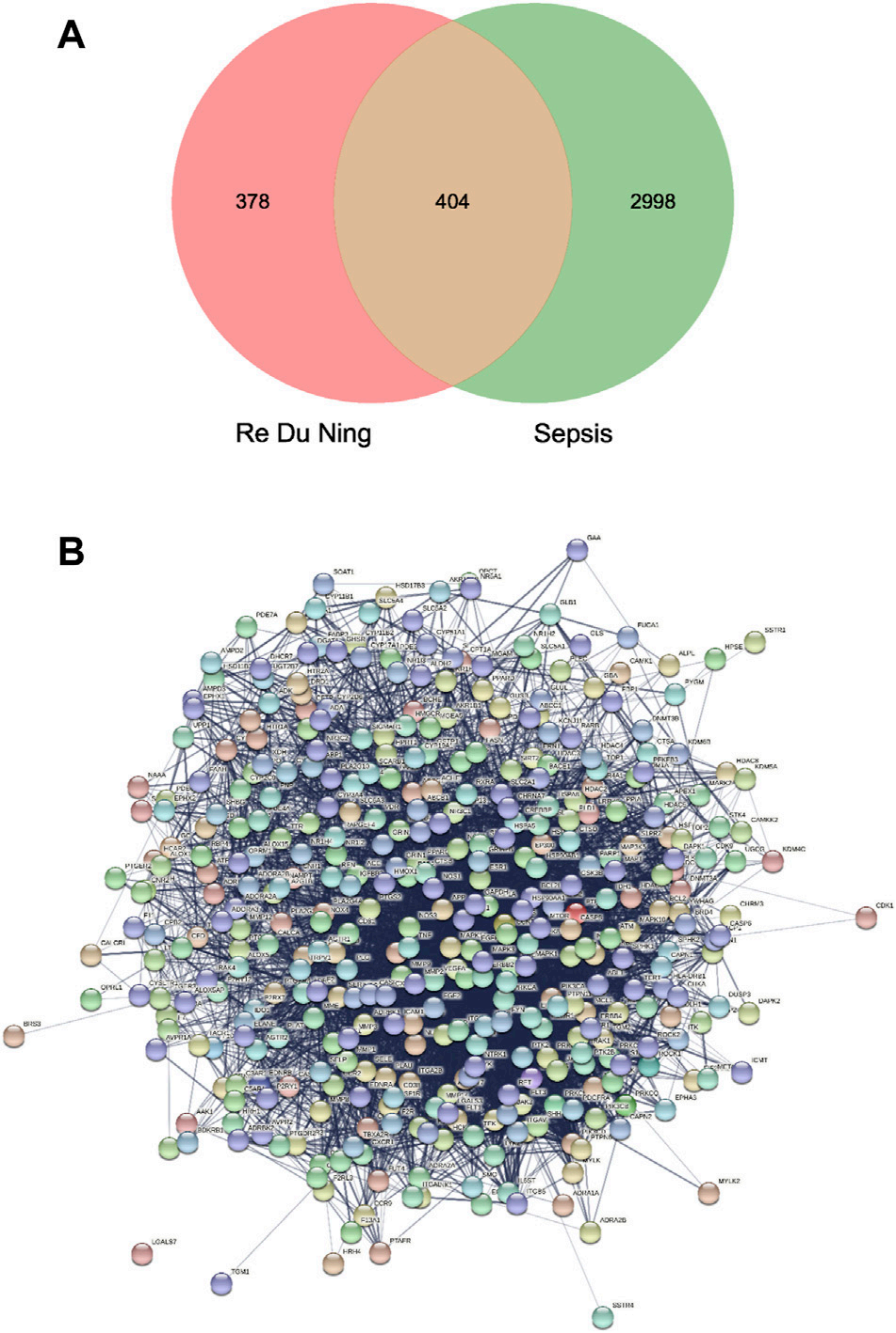
**(A)** Drug targets for RDN and sepsis targets. **(B)** PPI network of 404 overlapping gene symbols.

Venny 2.1 online software mapping tool platform was used to input 782 RDN drug targets and 3402 sepsis targets to draw Venny diagram. After intersection of the two, 404 drug-disease common targets were obtained (Figure 1A), indicating the effect of RDN on sepsis. There are 404 nodes and 7090 edges in the PPI network, and the average degree value is 35.1 (Figure 1B). The key target genes of the compound acting on the disease were screened according to the ranking of Degree value, as shown in Table 1.

**TABLE 1:** Shared key targets of sepsis and RDN.

Targets	Degree value
AKT1	221
TNF	216
IL6	197
GAPDH	196
MAPK3	165

### 3.4 Acquisition of RDN Targets for the Treatment of Sepsis and Construction of the “TCM - Component-Target-Disease” Network

**FIGURE 2:**
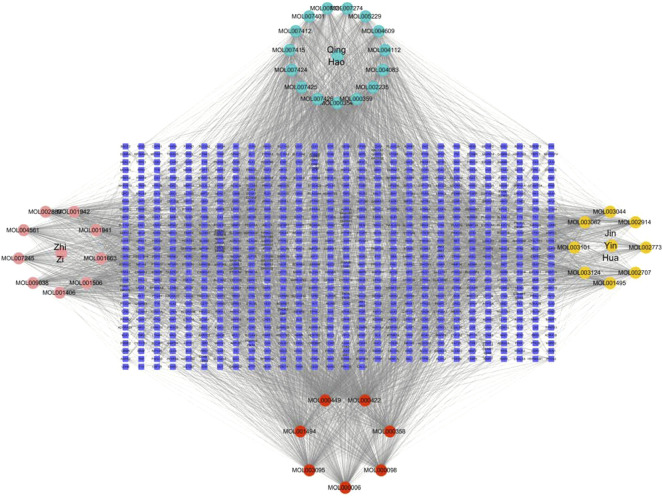
TCM-component-target-disease network. The red nodes represent the active ingredients in AMK; The pink nodes represent Gardenia gardenia, the green nodes represent Artemisia annua, and pink nodes represent Honeysuckle; The edges performed that nodes could interact with each other.

Cytoscape 3.7.2 software was applied to build a network diagram of “RDN-component-target-sepsis”. Potentially active components and drug-disease targets were input into Cytoscape software, and the interaction network of “RDN components target sepsis” was plotted (Figure 2).

### 3.5 Enrichment Analysis of Key Targets GO and KEGG

**FIGURE 3:**
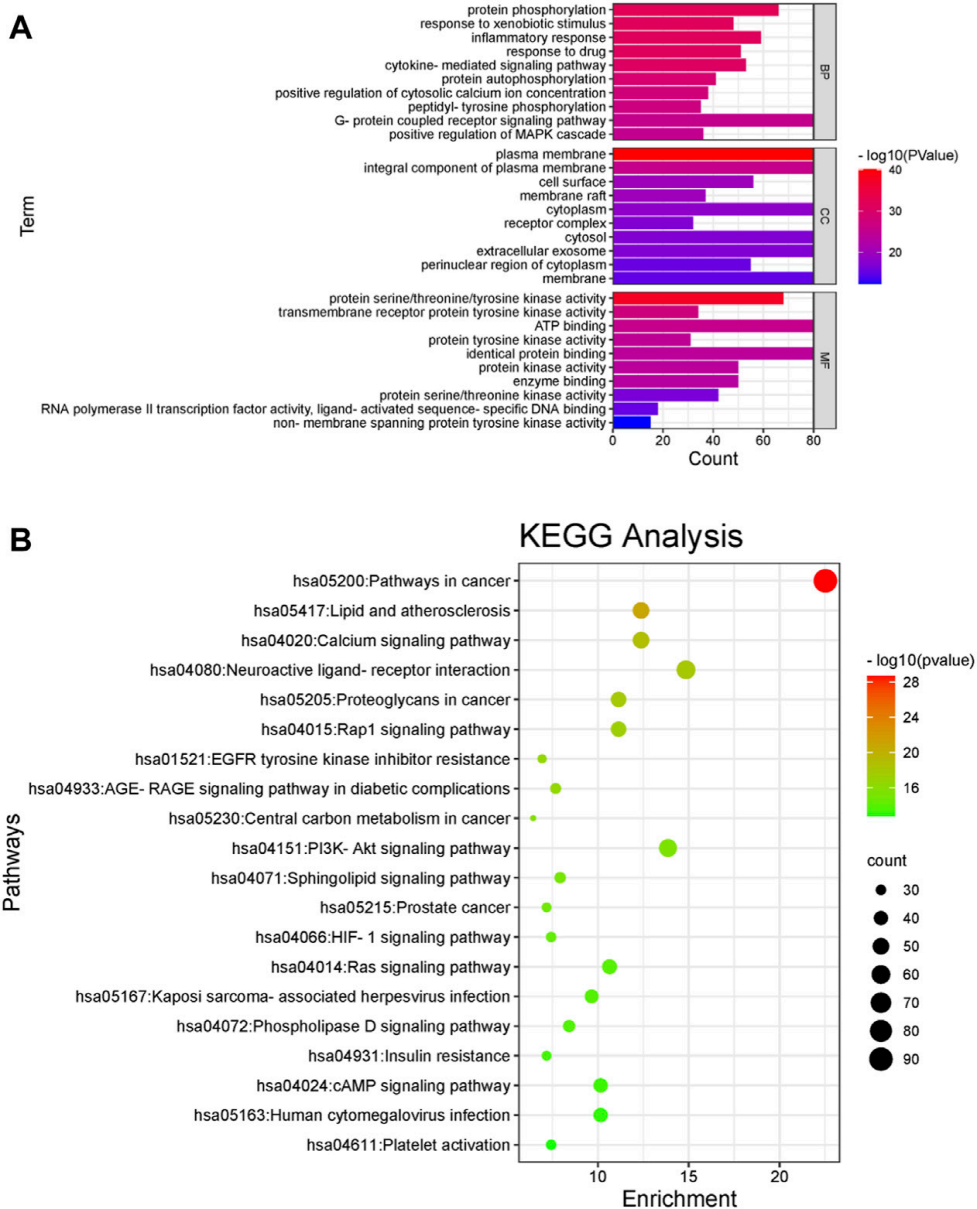
GO analyses and KEGG pathway enrichment analyses of the gene symbols associated with sepsis. **(A)** GO analyses of the gene symbols associated with sepsis. The *x*-axis represents significant enrichment in the counts of these terms. The *y*-axis represents the categories in the GO of the target genes (*p* < 0.05). The abscissa represents the number of targets; the left represents BP, CC and MF; the color represents *p*-value; the smaller the *p*-value, the redder the color is; the larger the *p*-value, the bluer it is. **(B)** KEGG pathway enrichment analyses of the gene symbols associated with sepsis. The *x*-axis represents the counts of the target symbols in each pathway; the *y*-axis represents the main pathways (*p* < 0.05). The abscissa represents the number of enriched genes, the left represents the name of pathway, and the color represents the *p*-value. The smaller the *p*-value, the more red the color is; the larger the *p*-value, the more green the color is.

As shown in Figures 3A, 1634 GO items were found to be enriched. There are 1231 BP, which mainly regulate and participate in protein phosphorylation, response to foreign stimuli, inflammatory reaction, drug reaction, protein autophosphorylation, peptidyl-Tyrosine phosphorylation, positive regulation of MAPK cascade, etc. There are 134 CC, mainly targeting plasma membrane, plasma membrane, cell surface, membrane raft, cytoplasm, receptor complex, cell foreign matter, perinuclear region of cytoplasm, etc. There are 269 MF, mainly in protein serine/threonine/tyrosine kinase activity, transmembrane receptor protein tyrosine kinase activity, the ATP binding protein kinase activity, across the membrane protein tyrosine kinase activity, RNA polymerase II transcription factor activity and ligand activation sequence-specific DNA binding, etc. The abscissa represents the number of targets; the left represents BP, CC and MF; the color represents *p*-value; the smaller the *p*-value, the redder the color is; the larger the *p*-value, the bluer it is.

As shown in Figures 3B, 87 signal pathways were enriched, largely in major cancer pathways, lipid, and atherosclerosis, calcium signaling pathway, stimulating neural tissue interaction, Rap1 signaling pathway, epidermal growth factor receptor (EGFR) tyrosine kinase inhibitor resistance, AGE-RAGE signaling pathway, cancer center carbon metabolism, and PI3K-AKT signaling pathway, etc.

### 3.6 Molecular Docking Analysis

**FIGURE 4:**
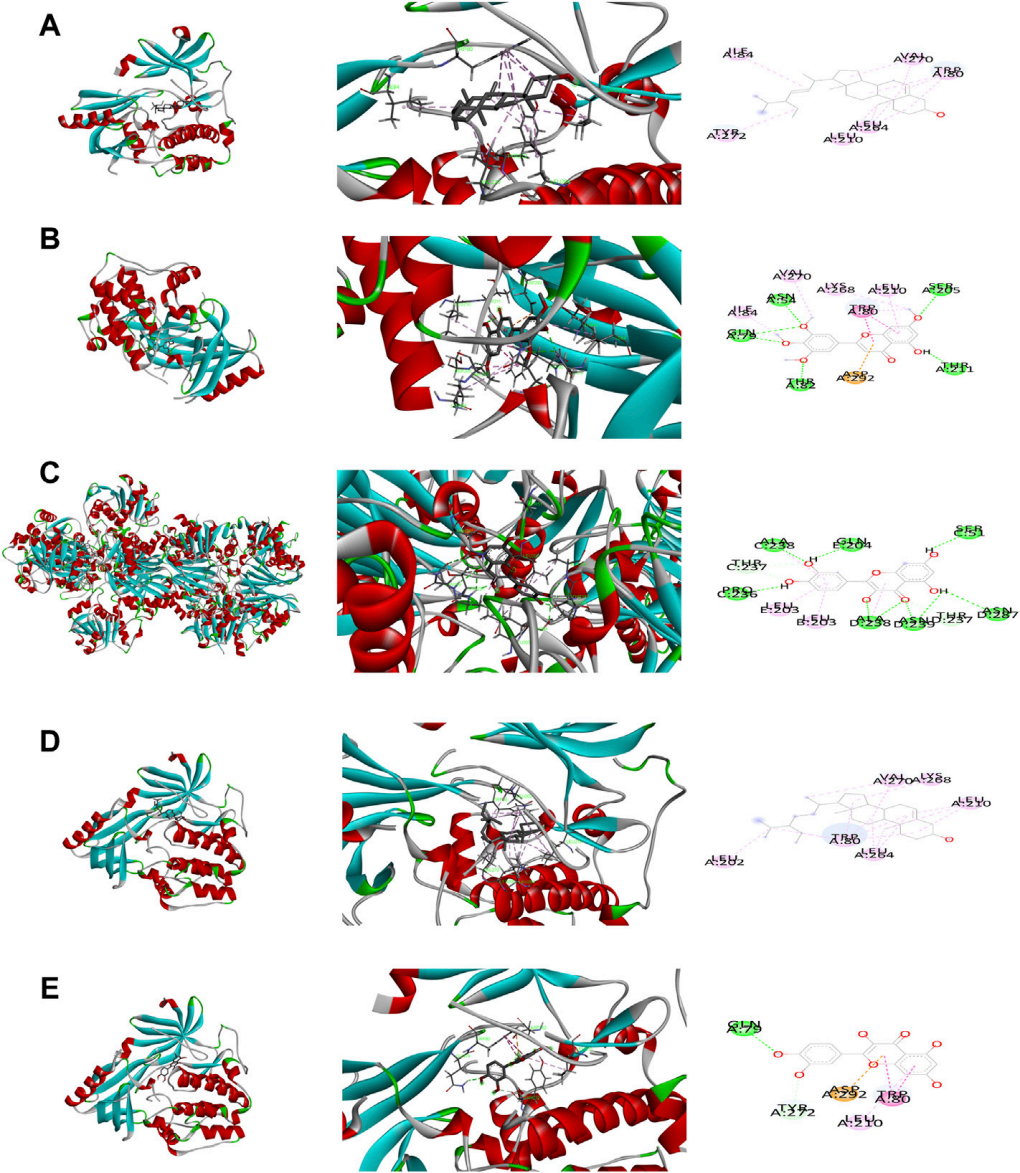
Binding studies of selected ingredients–targets interactions. **(A)** AKT with Stigmasterol; **(B)** AKT1 with Corymbosin; **(C)** GAPDH with quercetin; **(D)** AKT1 with beta-sitosterol; **(E)** AKT1 with quercetin.

Five key target structures and the five key active component structures were docked, and their Affinity (Kcal/mol) value represented the binding ability of the two. The lower the binding ability, the more stable the ligand and receptor binding. Finally, Discovery Studio software was used to analyze and observe the docking results. The key active ingredients Stigmasterol, Kaempferol, Quercetin, beta-sitosterol and Corymbosin were verified by molecular docking with the key targets AKT1, TNF, IL6, GAPDH and MAPK3. The results show that they all have good binding activity. The four results with the highest beating rank are selected and analyzed in charts. The results are shown in Table 2 and Figure 4.

**TABLE 2:** Molecular docking binding energy.

Number	Molecular	Protein	PDB id	Binding energey(kcal/Mol)
1	Stigmasterol	AKT1	4ejn	−11.3
2	Corymbosin	AKT1	4ejn	−9.9
3	Quercetin	GAPDH	6iq6	−9.8
4	beta-sitosterol	AKT1	4ejn	−9.5
5	Quercetin	AKT1	4ejn	−9.4
6	Kaempferol	AKT1	4ejn	−9.3
7	Quercetin	TNF	2e7a	−9.1
8	beta-sitosterol	TNF	2e7a	−9.1
9	Quercetin	MAPK3	2zoq	−9
10	Kaempferol	GAPDH	6iq6	−8.9
11	Corymbosin	GAPDH	6iq6	−8.9
12	Kaempferol	TNF	2e7a	−8.8
13	Kaempferol	MAPK3	2zoq	−8.6
14	beta-sitosterol	GAPDH	6iq6	−8.5
15	Corymbosin	TNF	2e7a	−8.5
16	Stigmasterol	GAPDH	6iq6	−7.8
17	Stigmasterol	MAPK3	2zoq	−7.7
18	beta-sitosterol	MAPK3	2zoq	−7.2
19	Stigmasterol	TNF	2e7a	−7.1
20	Kaempferol	IL6	7nxz	−7
21	Stigmasterol	IL6	7nxz	−6.9
22	Corymbosin	MAPK3	2zoq	−6.9
23	Quercetin	IL6	7nxz	−6.8
24	Corymbosin	IL6	7nxz	−6.7
25	beta-sitosterol	IL6	7nxz	−6.3

### 3.7 Pre-study to Determine Time Point and Concentration

**FIGURE 5:**
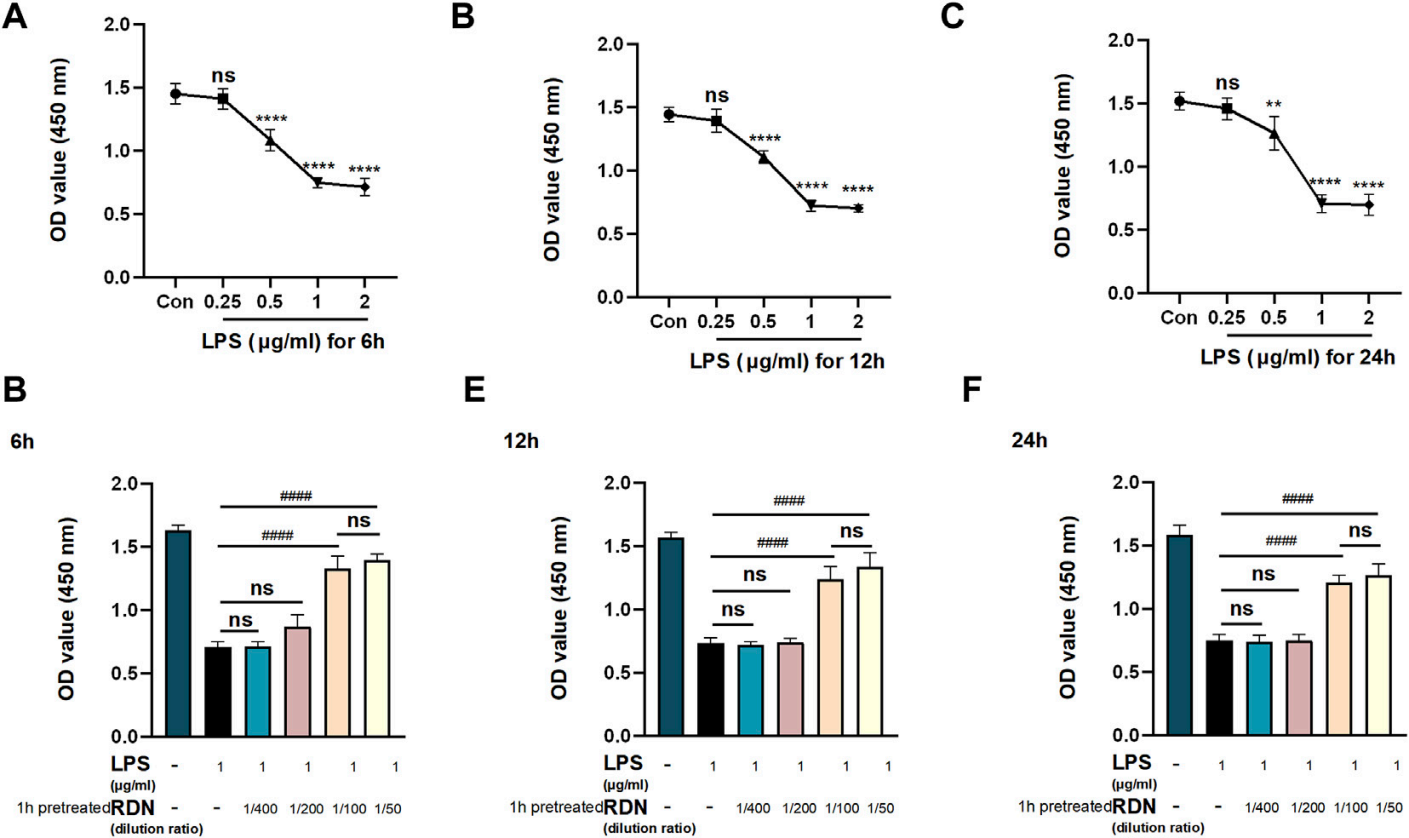
Pre-study to detect efficient time points and concentrations. CCK-8 was used to evaluate cell viability to find efficient concentrations and key points. HUVECs were treated with LPS (0.25 μg/ml, 0.5 μg/ml, 1 μg/ml, 2 μg/ml) for 6 h **(A)**, 12 h **(B)**, and 24 h **(C)** to find efficient concentration and time point of LPS. HUVECs were stimulated with LPS (and RDN) and cultured for 6 h **(D)**, 12 h **(E)**, and 24 h **(F)** to find efficient concentration and time point of RDN. The RDN group was pretreated with cells diluted into different concentrations of RDN injection (dilution in 1/400, 1/200, 1/100, 1/50) for 1 h. Data are presented as mean ± SD (*n* = 3 per group) of the representative data from three independent experiments; **p < 0.01, ****p < 0.001, ^####^ *p* < 0.001. The asterisk (*) represents the group is statistically different from the Con group.

In order to verify the reliability of the main effective targets and molecular mechanism of RDN in the treatment of sepsis predicted by network pharmacology experiments, LPS was selected to mimic septic cell model and RDN injection was selected as intervention. First, the effective time point and concentrations of LPS and were determined by CCK-8. LPS group was treated with different concentrations of LPS (0.25 μg/ml, 0.5 μg/ml, 1 μg/ml, 2 μg/ml) for 6 h, 12 h, and 24 h. Control (Con) group was not treated with intervention. As shown in Figures 5A–C, the OD value enhanced in LPS group from each time point compared to the same time points in the Con group (*p* < 0.05). The OD value in LPS group was not significantly different from that in Con group when LPS was injected at 0.25 μg/ml (*p* > 0.05). The OD value in LPS group was significantly higher than that in Con group when LPS was injected at 0.5 μg/ml, 1 μg/ml, and 2 μg/ml (*p* < 0.05). We selected 1 μg/ml LPS for the following experiment. Next, we carried out the study with different concentrations of RDN injection (dilution in 1/400, 1/200, 1/100, 1/50) and LPS to identify effective time point and RDN concentration. The LPS + RDN group was pretreated with cells diluted into different concentrations of RDN injection (dilution in 1/400, 1/200, 1/100, 1/50) for 1h, and then stimulated with 1 μg/ml LPS for 6h, 12h and 24 h to simulate sepsis. As shown in Figures 5D–F, RDN could dramatically alleviate apoptosis level in LPS group from each time point (*p* < 0.05). To make the study more operational, we chose 6 h as the efficient time point.

### 3.8 RDN Could Interfere With PI3K-AKT Signaling Pathway in LPS-Induced Cells

**FIGURE 6:**
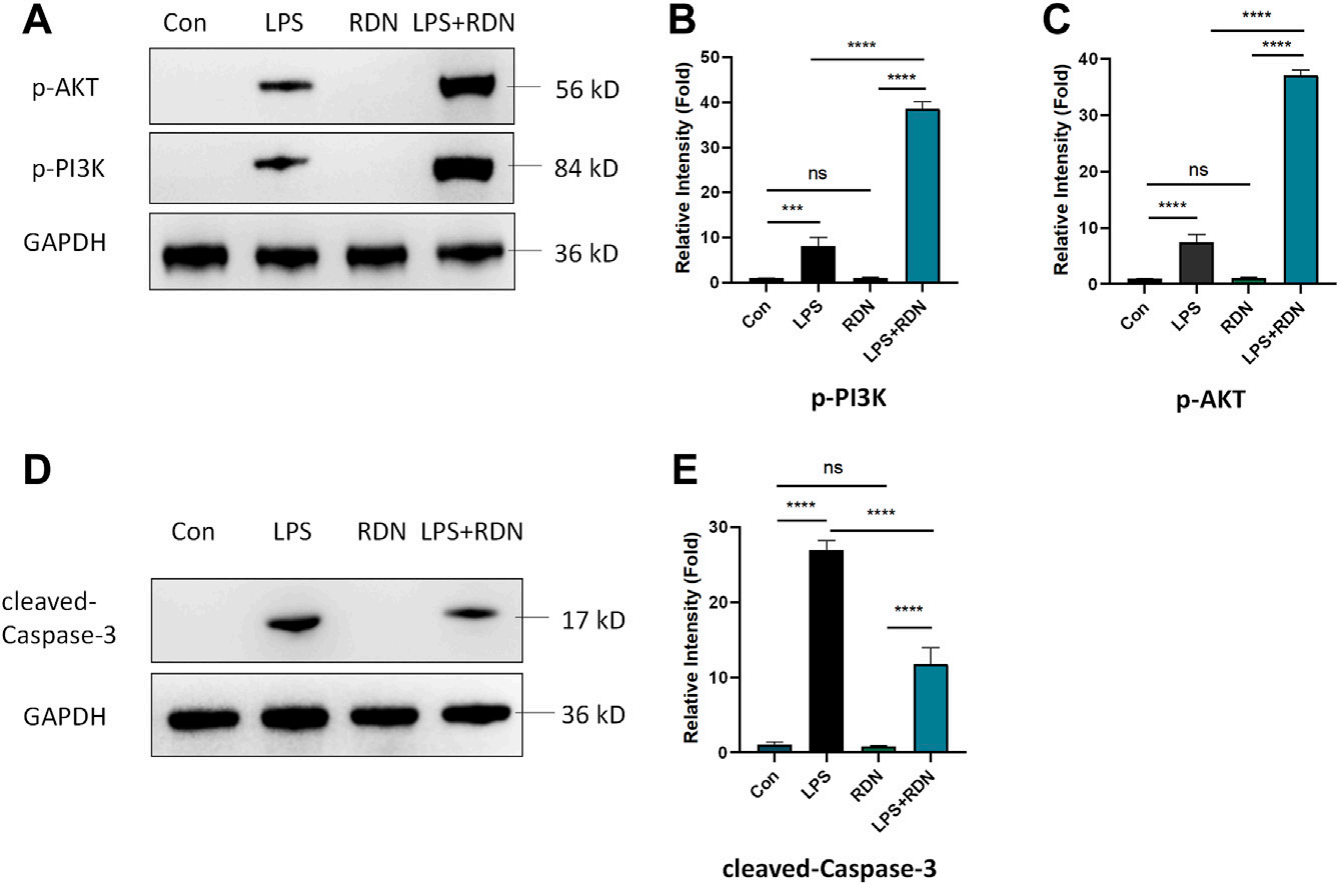
RDN could interfere with PI3K-AKT-Caspase-3 signaling pathway in LPS-induced HUVECs. **(A-C)** Western blot was employed to detect the effect of RDN on PI3K-AKT signaling pathway. **(D,E)** The protein expression of cleaved-Caspase-3 in HUVECs was analyzed by western blotting. The results were quantified by densitometry. Data are presented as mean ± SD (*n* = 3 per group) of the representative data from three independent experiments; ***p < 0.005, ****p < 0.001.

To further verify the effect of RDN on PI3K-AKT signaling pathway, western blot was employed to detect the results of network pharmacology analysis. As shown in Figures 6A–C, compared with Con group, the protein expression level of p-AKT and p-PI3K were enhanced in LPS group (*p* < 0.005). Compared with LPS group, the protein expression level of p-AKT and p-PI3K were critically increased in LPS + RDN group (*p* < 0.001). There is no statistical difference between Con group and RDN group (*p* > 0.05).

### 3.9 RDN Could Alleviate Apoptosis Level in LPS-Induced Cells

**FIGURE 7:**
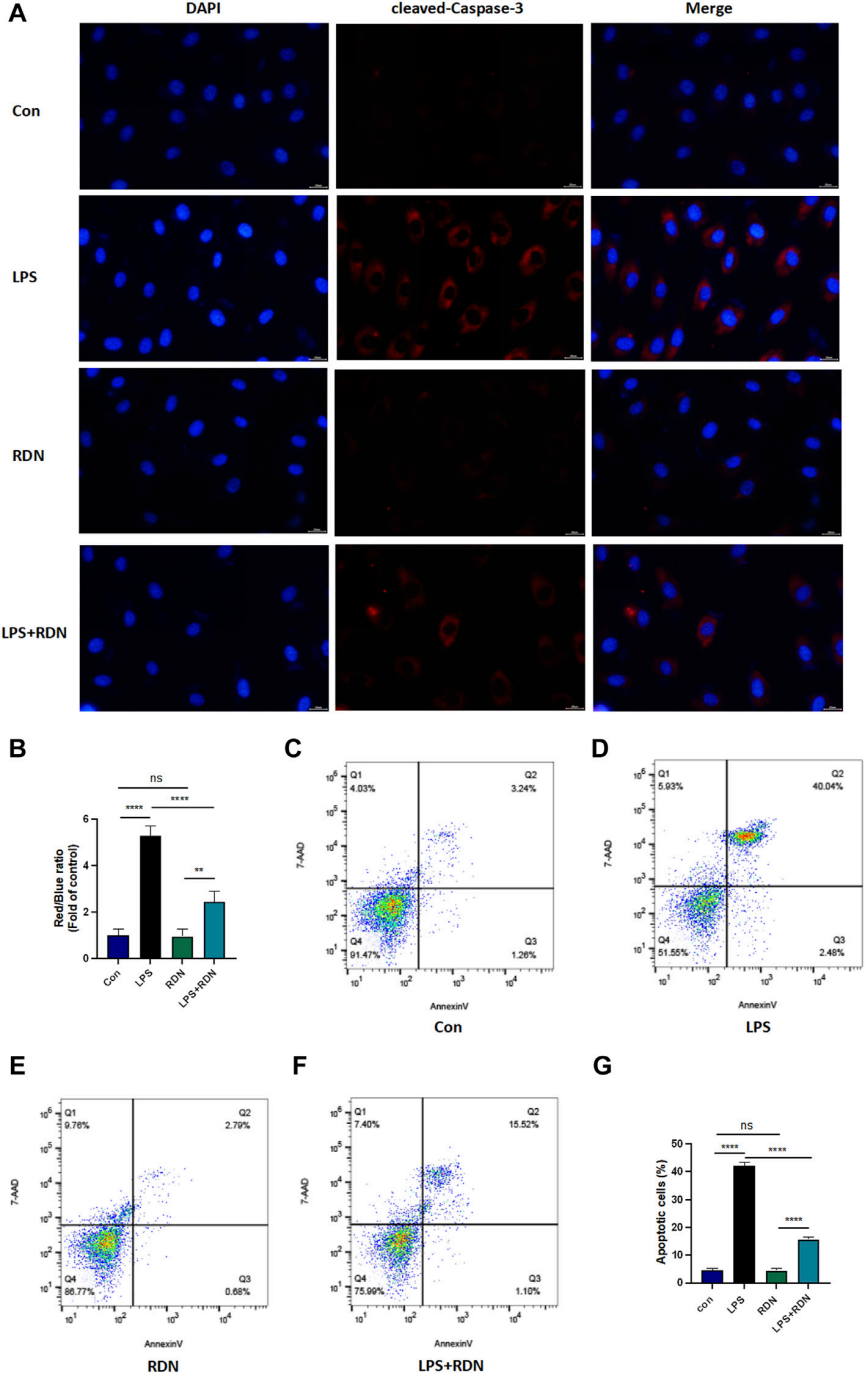
RDN could alleviate LPS-induced HUVECs apoptosis. **(A,B)** Immunofluorescence was performed to detect the level of apoptosis related protein. Magnification×40, scale bar 20 μm. **(C-G)** Flow cytometry was employed to detect apoptosis rate. Cells were equally divided into the Con group, LPS group, RDN group, LPS + RDN group and evaluated the proportion of apoptotic cells (Q2+Q3) in each group. Data are presented as mean ± SD (*n* = 3 per group) of the representative data from three independent experiments; **p < 0.01, ****p < 0.001.

Western blot was performed to detect the level of apoptosis-related protein. As shown in Figures 6D,E, compared with Con group, the protein expression level of cleaved-Caspase-3 was significantly increased in LPS group (*p* < 0.001). Compared with LPS group, the protein expression level of cleaved-Caspase-3 was critically decreased in LPS + RDN group (*p* < 0.001). Immunofluorescence was also used to investigate the level of apoptosis-related protein. As shown in Figures 7A,B, compared with Con group, the ratio of cleaved-Caspase-3 was significantly increased in LPS group (*p* < 0.001). Compared with LPS group, the protein expression of cleaved-Caspase-3 was critically decreased in LPS + RDN group (*p* < 0.001). There is no statistical difference between Con group and RDN group (*p* > 0.05).

Flow cytometry was performed to show apoptosis level. 7-AAD and Annexin-V assay Q2 + Q3 were used to exhibit the apoptosis rate. As shown in Figures 7C–G, LPS could significantly increased apoptosis level (*p* < 0.001), RDN could critically alleviate it (*p* < 0.001), and the proportions of apoptosis cells were 4.73 ± 0.416, 42.24 ± 1.044, 4.44 ± 0.711, and 15.597 ± 0.662 in Con group, LPS group, RDN group, and LPS + RDN group respectively.

## 4 Discuss

Sepsis 3.0 released in 2016 considers the occurrence of multiple organ dysfunction syndrome (MODS) as the key link in sepsis (Seymour et al., 2016). However, organ injuries are difficult to reverse once they occur. Despite a large number of clinical and basic studies on sepsis, the incidence and mortality of sepsis remain high (Chiu and Legrand, 2021). Based on this, In 2020, Chinese emergency medicine experts put forward the conception of “prevention and blocking sepsis” and carried out “Preventing Sepsis Campaign China” to achieve early prevention, early detection and early intervention. They proposed that TCM could be used to prevent and block sepsis before organ injury (Wang et al., 2020a).

RDN, as a representative of traditional Chinese patent medicine, is the treatment of upper respiratory tract infection caused by high fever, mild wind chill, head and body pain, cough and yellow phlegm and other diseases (Wu et al., 2020). Nowadays, the functions of clearing heat, dispersing wind and detoxifying are gradually applied to other diseases, such as COVID-19 (Ma et al., 2021), monocytic angina (Xu, GAO, Chen), hand-foot-and-mouth disease (Dai and Xia, 2021), and acute cholecystitis (Chen et al., 2021). It has also been proved that RDN plays a prominent role in the prevention and treatment of sepsis (Zheng, 2019b). Clinical studies have pointed out that timely and appropriate use of RDN injection could shorten the time of disease treatment, reduce the probability of organ function damage, and effectively improve sepsis patient outcomes (Wang and Li, 2015). Basic studies have also argued that RDN and its components could alleviate inflammatory response and oxidative stress, and improve sepsis in a variety of ways, thus relieving organ damage in patients with sepsis, but the mechanism is still unclear. Some authors evidenced that RDN can inhibit NF-κB pathway in LPS-induced acute lung injury rat model to prevent pulmonary neutrophil infiltration, reduce MPO activity, and improve inflammatory response (Tang et al., 2014; Yang et al., 2021). The three TCM components of RDN also have anti-inflammatory effects respectively. Yang JH et al. found that artemisia annua extract can reduce TNF-α/IFN-γ -induced proinflammatory factors *in vitro* (Kim et al., 2013). Feng J et al. found that the extract cynaroside of Lonicerae japonicae could improve multiple organ functional injury by reversing macrophage polarization, and the extract HS-23 could also reduce CLP-induced CD4(+) and CD8(+) T cell apoptosis, inhibit the internal and external apoptotic pathways in spleen, and improve the immune suppression induced by sepsis (Feng et al., 2021). Su Q et al. proposed that Gardenia jasminoides could attenuate inflammatory response and apoptosis by regulating mir-145 and MEK/ERK pathways *in vitro* experiments (Su et al., 2018). Cui Y et al. also showed that Gardenia water decoction had protective effect on LPS-induced intestinal mucosal injury by reducing oxidative stress level (Cui et al., 2019).

On the basis of the theory of pharmacology and biological multidisciplinary cross (Sharma et al., 2018), with “compound - gene - targets - disease” perspective to reveal complex network, we used the network pharmacology to study multiple components and multiple targets of TCM, to predict potential pharmacological mechanism of TCM for the prevention and blocking sepsis. In our study, an interactive network diagram of “component-target-disease” was constructed by network pharmacology analysis method to comprehensively and systematically sorted out the mechanism of RDN in the treatment of sepsis. We found that AKT1 ranked highest in degree value among shared targets of disease drugs. The key active ingredients were verified by molecular docking with AKT1, TNF, IL-6, GAPDH and MAPK3, the top five key targets of degree value, respectively. The smaller binding energy, the better docking effect. The results showed that AKT1 had the best binding activity with stigmasterol. And four of the five results with the best docking results were related to AKT1. KEGG enrichment analysis of shared targets showed that RDN could alleviate sepsis mainly involving PI3K/AKT pathway.

Studies suggested that the PI3K/AKT pathway could play a key role in apoptosis and autophagy, and may affect the development of sepsis. Shang X et al. showed that activation of the PI3K/AKT pathway could enhance autophagy, thereby down-regulating the inflammatory response and alleviating myocardial damage in sepsis (Shang et al., 2019). Qu Y et al. found that activation of PI3K/AKT pathway can down-regulate apoptosis level and improve septic AKI in sepsis rat model (Qu et al., 2020). Santos DMD et al. found that activation of the PI3K/AKT pathway can reduce the impact of sepsis on the spleen, liver, kidney, and other organs by down-regulating the levels of inflammatory response, and oxidative stress (Santos et al., 2021). Other scholars proposed that inhibition of PI3K/AKT pathway could reduce oxidative stress levels and up-regulate apoptosis levels *in vitro* (Li et al., 2018; Li et al., 2019).

To sum up, the PI3K-AKT pathway is closely related to sepsis cell apoptosis. Our previous study have proposed that apoptosis of renal tubular epithelial cells plays an important role in sepsis acute kidney injury, and the level of apoptosis-related proteins and apoptosis rate were increased in septic cell models (Wang et al., 2020b). Therefore, we mainly focused on the role of RDN in sepsis through PI3K-AKT pathway mediated apoptosis.

Vascular endothelial cells are the most important effector cells in coagulation initiation and inflammatory activation. Sepsis is the host’s response to infection that causes life-threatening organ dysfunction. Therefore, the activation and dysfunction of vascular endothelial cells are the central link in the progression and deterioration of sepsis. HUVECs, which could (in theory) be passed on indefinitely, has been widely used in investigating apoptosis in sepsis (Lee et al., 2014; van der Slikke et al., 2021; Fang et al., 2022). In our study, we applied LPS-induced HUVECs as the septic endothelial cell model to verify our results of network pharmacology. Although septic cell model is still controversial, stimulation of cells with LPS is currently the common method to simulate the cell circumstance of sepsis (Deng et al., 2018). We observed the PI3K-AKT signaling pathway by western blot, detected cell apoptosis rate by flow cytometry, and investigated apoptosis-related protein expression level by immunofluorescence and western blot. Compared with Con group, cell apoptosis rate and apoptosis-related protein expression level were dramatically enhanced in LPS group. It was found that LPS could activated the PI3K-AKT signaling pathway. This maybe because PI3K-AKT signaling pathway also plays other roles in LPS-induced infection in addition to apoptosis. Compared with LPS group, the PI3K-AKT signaling pathway was significantly activated in the RDN group, paralleled with decreased cell apoptosis rate and apoptosis-related protein expression level. Combined with the results of network pharmacology analysis, it was illustrated that inhibiting the PI3K/AKT signaling pathway could activate apoptosis in sepsis, while RDN may activate this pathway by targeting AKT1, thus improving apoptosis and slowing down endothelial cell injury.

## 5 Limitation

As with most studies, the design of the current study is apt to limitations. This study sorely investigated the potential mechanism of RDN on LPS-induced HUVECs apoptosis through PI3K-AKT pathway, but fell short of verifying the core target AKT1. However, we have demonstrated the binding ability of AKT1 and RDN in Molecular docking analysis, so this limitation will not cause a very large bias in the results of the study. We will continue to explore the mechanism by knocking out AKT1 *in vivo* and *in vitro*.

## 6 Conclusion

In conclusion, this study applied the method of network pharmacology to study the complex molecular network relationship between RDN and sepsis, and found that RDN may regulate core genes such as AKT1, IL-6, TNF, and PTGS2 through active ingredients such as Stigmasterol, Kaempferol, Quercetin, beta-sitosterol, and Corymbosin, thus improving the endothelial cell damage caused by LPS. AKT1 may be the core target, and PI3K-AKT pathway may be the core pathway of RDN to improve the apoptosis of endothelial cells caused by LPS. This study may provide a new clue for molecular mechanism of RDN in the treatment of sepsis, and provide a certain basis for its clinical application.

## Data Availability

The existing datasets are available in a publicly accessible repository. Publicly available datasets were analyzed in this study. This data can be found here: TCMSP (https://tcmspw.com/tcmsp.php/), GeneCards human genome database (https://www.genecards.org/), DisGenet database (https://www.disgenet.org/), and OMIM database (https://omim.org/).

## References

[B1] Bruno R. R. Wernly B. Mamandipoor B. Rezar R. Binnebössel S. Baldia P. H. (2021). ICU-mortality in Old and Very Old Patients Suffering from Sepsis and Septic Shock. Front. Med. (Lausanne) 8, 697884. 10.3389/fmed.2021.697884 34307423 PMC8299710

[B2] Chen J. J. Zhang H. Y. Qu Y. Cao X. Song X. (2021). Effect of Reduening Injection Adjuvant Therapy on Inflammatory Indexes and Immune Function in Patients with Acute Cholecystitis [J]. J. Clin. Emerg. 22 (08), 540–543.

[B3] Chiu C. Legrand M. (2021). Epidemiology of Sepsis and Septic Shock. Curr. Opin. Anaesthesiol. 34 (2), 71–76. 10.1097/ACO.0000000000000958 33492864

[B4] Cui Y. Wang Q. Wang M. Jia J. Wu R. (2019). Gardenia Decoction Prevent Intestinal Mucosal Injury by Inhibiting Pro-inflammatory Cytokines and NF-Κb Signaling. Front. Pharmacol. 10, 180. 10.3389/fphar.2019.00180 30983991 PMC6447716

[B5] Dai S. S. Xia C. (2021). Effect of Reduining Combined with Five-Dimensional Lysine Granules on Serum cTnI and CK-MB Levels in the Treatment of Hand-Foot-Mouth Disease [J]. Chin. J. Traditional Chin. Med., 1–7.

[B6] Deng M. Tang Y. Li W. Wang X. Zhang R. Zhang X. (2018). The Endotoxin Delivery Protein HMGB1 Mediates Caspase-11-dependent Lethality in Sepsis. Immunity 49 (4), 740–e7. 10.1016/j.immuni.2018.08.016 30314759 PMC6300139

[B7] Fang X. Duan S. F. Hu Z. Y. Wang J. J. Qiu L. Wang F. (2022). Inhibition of Matrix Metalloproteinase-8 Protects against Sepsis Serum Mediated Leukocyte Adhesion. Front. Med. (Lausanne) 9, 814890. 10.3389/fmed.2022.814890 35145983 PMC8821815

[B8] Feng J. Liu Z. Chen H. Zhang M. Ma X. Han Q. (2021). Protective Effect of Cynaroside on Sepsis-Induced Multiple Organ Injury through Nrf2/HO-1-dependent Macrophage Polarization. Eur. J. Pharmacol. 911, 174522. 10.1016/j.ejphar.2021.174522 34560076

[B9] Jarczak D. Kluge S. Nierhaus A. (2021). Sepsis-Pathophysiology and Therapeutic Concepts. Front. Med. (Lausanne) 8, 628302. 10.3389/fmed.2021.628302 34055825 PMC8160230

[B10] Jiang C. Zhong R. Zhang J. Wang X. Ding G. Xiao W. (2019). Reduning Injection Ameliorates Paraquat-Induced Acute Lung Injury by Regulating AMPK/MAPK/NF-κB Signaling. J. Cell Biochem. 120 (8), 12713–12723. 10.1002/jcb.28540 30861187

[B11] Kim H. G. Yang J. H. Han E. H. Choi J. H. Khanal T. Jeong M. H. (2013). Inhibitory Effect of Dihydroartemisinin against Phorbol Ester-Induced Cyclooxygenase-2 Expression in Macrophages. Food Chem. Toxicol. 56, 93–99. 10.1016/j.fct.2013.02.017 23429041

[B12] Lee W. Yoo H. Ku S. K. Kim S. W. Bae J. S. (2014). Raftlin: a New Biomarker in Human Sepsis. Inflammation 37 (3), 706–711. 10.1007/s10753-013-9788-7 24317805

[B13] Li M. Ye J. Zhao G. Hong G. Hu X. Cao K. (2019). Gas6 Attenuates Lipopolysaccharideinduced TNFalpha Expression and Apoptosis in H9C2 Cells through NFkappaB and MAPK Inhibition via the Axl/PI3K/Akt Pathway[J]. Int. J. Mol. Med. 44 (3), 982–994. 31524235 10.3892/ijmm.2019.4275 PMC6657963

[B14] Li S. T. Dai Q. Zhang S. X. Liu Y. J. Yu Q. Q. Tan F. (2018). Ulinastatin Attenuates LPS-Induced Inflammation in Mouse Macrophage RAW264.7 Cells by Inhibiting the JNK/NF-κB Signaling Pathway and Activating the PI3K/Akt/Nrf2 Pathway. Acta Pharmacol. Sin. 39 (8), 1294–1304. 10.1038/aps.2017.143 29323338 PMC6289329

[B15] Luo S. Gan L. Liu S. Zhong L. Chen M. Zhang H. (2022). The Synergistic Reduning and Cefmetazole Sodium Treatment of Severe Pneumonia Is Mediated by the AhR-Src-STAT3 Pathway. J. Thorac. Dis. 14 (2), 474–493. 10.21037/jtd-22-126 35280469 PMC8902113

[B16] Ma Q. Xie Y. Wang Z. Lei B. Chen R. Liu B. (2021). Efficacy and Safety of ReDuNing Injection as a Treatment for COVID-19 and its Inhibitory Effect against SARS-CoV-2. J. Ethnopharmacol. 279, 114367. 10.1016/j.jep.2021.114367 34174375 PMC8223030

[B17] Qu Y. Sun Q. Song X. Jiang Y. Dong H. Zhao W. (2020). Helix B Surface Peptide Reduces Sepsis-Induced Kidney Injury via PI3K/Akt Pathway. Nephrol. Carlt. 25 (7), 527–534. 10.1111/nep.13683 31778269

[B18] Santos D. M. D. Da Silva E. A. P. Oliveira J. Y. S. Marinho Y. Y. M. Santana I. R. Heimfarth L. (2021). The Therapeutic Value of Hydralazine in Reducing Inflammatory Response, Oxidative Stress, and Mortality in Animal Sepsis: Involvement of the PI3K/AKT Pathway. Shock 56 (5), 782–792. 10.1097/SHK.0000000000001746 33555842

[B19] Scheer C. S. Kuhn S. O. Fuchs C. Vollmer M. Modler A. Brunkhorst F. (2019). Do Sepsis-3 Criteria Facilitate Earlier Recognition of Sepsis and Septic Shock? A Retrospective Cohort Study. Shock 51 (3), 306–311. 10.1097/SHK.0000000000001177 30422118

[B20] Seymour C. W. Liu V. X. Iwashyna T. J. Brunkhorst F. M. Rea T. D. Scherag A. (2016). Assessment of Clinical Criteria for Sepsis: For the Third International Consensus Definitions for Sepsis and Septic Shock (Sepsis-3). JAMA 315 (8), 762–774. 10.1001/jama.2016.0288 26903335 PMC5433435

[B21] Shang X. Lin K. Yu R. Zhu P. Zhang Y. Wang L. (2019). Resveratrol Protects the Myocardium in Sepsis by Activating the Phosphatidylinositol 3-Kinases (PI3K)/AKT/Mammalian Target of Rapamycin (mTOR) Pathway and Inhibiting the Nuclear Factor-Κb (NF-Κb) Signaling Pathway. Med. Sci. Monit. 25, 9290–9298. 10.12659/msm.918369 31806860 PMC6911307

[B22] Sharma A. Angulo-Bejarano P. I. Madariaga-Navarrete A. Oza G. Iqbal H. M. N. Cardoso-Taketa A. (2018). Multidisciplinary Investigations on Galphimia Glauca: A Mexican Medicinal Plant with Pharmacological Potential. Molecules 23 (11). 10.3390/molecules23112985 PMC6278297 30445751

[B23] Su Q. Yao J. Sheng C. (2018). Geniposide Attenuates LPS-Induced Injury via Up-Regulation of miR-145 in H9c2 Cells. Inflammation 41 (4), 1229–1237. 10.1007/s10753-018-0769-8 29611016

[B24] Tang L. P. Xiao W. Li Y. F. Li H. B. Wang Z. Z. Yao X. S. (2014). Anti-inflammatory Effects of Reduning Injection on Lipopolysaccharide-Induced Acute Lung Injury of Rats. Chin. J. Integr. Med. 20 (8), 591–599. 10.1007/s11655-014-1758-x 24916807 PMC7101712

[B25] van der Slikke E. C. Star B. S. van Meurs M. Henning R. H. Moser J. Bouma H. R. (2021). Sepsis Is Associated with Mitochondrial DNA Damage and a Reduced Mitochondrial Mass in the Kidney of Patients with Sepsis-AKI. Crit. Care 25 (1), 36. 10.1186/s13054-020-03424-1 33494815 PMC7831178

[B26] Wang S. Y. Li G. (2015). Reduning Injection in Pediatric Clinical Application [J]. Glob. J. traditional Chin. Med. 8 (S1), 100.

[B27] Wang Z. Chen W. Li Y. Zhang S. Lou H. Lu X. (2021). Reduning Injection and its Effective Constituent Luteoloside Protect against Sepsis Partly via Inhibition of HMGB1/TLR4/NF-κB/MAPKs Signaling Pathways. J. Ethnopharmacol. 270, 113783. 10.1016/j.jep.2021.113783 33421596

[B28] Wang Z. Wang L. Cao C. Jin H. Zhang Y. Liu Y. (2020). Heparin Attenuates Histone-Mediated Cytotoxicity in Septic Acute Kidney Injury. Front. Med. (Lausanne) 7, 586652. 10.3389/fmed.2020.586652 33344474 PMC7738632

[B29] Wang Z. Wei J. Zhu H. D. (2020). Expert Consensus on Early Prevention and Prevention of Emergency Sepsis in China[J]. Chin. Emerg. Med. 40 (07), 577–588.

[B30] Wu P. Jiang Y. Zheng W. Lai Y. Huang X. Zhuang H. (2020). Molecular Mechanism of Reduning Injection in the Treatment of Dengue Fever Based on Network Pharmacology [J]. J. Guangzhou Univ. Traditional Chin. Med. 39 (01), 143–151.

[B31] Xie J. Wang H. Kang Y. Zhou L. Liu Z. Qin B. (2020). The Epidemiology of Sepsis in Chinese ICUs: A National Cross-Sectional Survey. Crit. Care Med. 48 (3), e209–e218. 10.1097/CCM.0000000000004155 31804299

[B32] Xu X. M. Gao J. Chen B. Q. Clinical Effect of Reduining Combined with Adenosine in the Treatment of Children with Infectious Mononucleosis and its Effect on T Lymphocyte Subsets and Inflammatory Factors [J]. Chin. J. Med. 16 (12), 1874–1877.

[B33] Yang C. Song C. Liu Y. Qu J. Li H. Xiao W. (2021). Re-Du-Ning Injection Ameliorates LPS-Induced Lung Injury through Inhibiting Neutrophil Extracellular Traps Formation. Phytomedicine 90, 153635. 10.1016/j.phymed.2021.153635 34229173 PMC8213523

[B34] Zhang G. Zhao J. He L. Yan S. Zhuo Z. Zheng H. (2013). Reduning Injection for Fever, Rash, and Ulcers in Children with Mild Hand, Foot, and Mouth Disease: a Randomized Controlled Clinical Study. J. Tradit. Chin. Med. 33 (6), 733–742. 10.1016/s0254-6272(14)60005-4 24660604

[B35] Zheng L. B. (2019). Effect Observation of Reduening Injection in Adjuvant Treatment of Early Sepsis Patients and its Influence on Inflammatory Factors [J]. China Sci. Technol. traditional Chin. Med. 26 (02), 172–175.

[B36] Zheng L. (2019). Effect Observation of Reduning Injection in Adjuvant Treatment of Early Sepsis Patients and its Influence on Inflammatory Factors[J]. Chin. Med. Sci. Technol. 26 (02), 172–175.

[B37] Zhou W. Lai X. Wang X. Yao X. Wang W. Li S. (2021). Network Pharmacology to Explore the Anti-inflammatory Mechanism of Xuebijing in the Treatment of Sepsis. Phytomedicine 85, 153543. 10.1016/j.phymed.2021.153543 33799226

[B38] Zhou Y. Sun F. Wu W. Sun L. Niu C. Cheng Z. (2016). Effect of Reduning Injection on ARDS Patients with Sepsis[J]. Shandong Med. 56 (12), 74–76.

